# Continued collaboration of *ex situ* and *in situ* programs is critical for the genetic sustainability of the endangered *Rana pretiosa*

**DOI:** 10.1038/s41598-025-01483-4

**Published:** 2025-05-22

**Authors:** Briar Hunter, Anne-Laure Ferchaud, Eric Normandeau, Kendra Morgan, Arne Mooers, Gabriela Mastromonaco, David Lesbarrères

**Affiliations:** 1 Department of Biology, Laurentian University, Sudbury, ON Canada; 2 Institut de Biologie Intégrative Et Des Systèmes (IBIS), Université Laval, Québec, Canada; 3 Parks Canada, Office of the Chief Ecosystem Scientist, Protected Areas Establishment and Conservation Directorate, Québec, QC Canada; 4 Ministry of Water, Land and Resource Stewardship (South Coast), Surrey, BC Canada; 5 Biological Sciences, Simon Fraser University, Burnaby, BC Canada; 6 Reproductive Science, Toronto Zoo, Toronto, ON Canada; 7 Environment and Climate Change Canada, Ottawa, ON Canada

**Keywords:** Animal breeding, Conservation genomics, Conservation biology

## Abstract

Retaining sufficient genetic variation for both short and long-term sustainability is a chief aim of ex situ programs for threatened species. Conservation breeding and reintroduction programs exist but oftentimes little is known about the genetic variation of in situ or ex situ populations. We collected genetic samples from both wild and zoo populations of Canada’s most endangered anuran, the Oregon Spotted Frog (*Rana pretiosa*) to compare genetic diversity (observed and expected heterozygosity), inbreeding coefficients (F_IS_), effective population sizes (Ne) and population structure using single-nucleotide polymorphisms (SNPs). We found low diversity in situ and lower diversity ex situ, with positive inbreeding coefficients indicating assortative mating in both wild and zoo populations. Ex situ breeding programs that allowed free mate choice retained more genetic variation compared to those where breeding groups were pre-determined. Mixed source zoo populations were less differentiated from their wild source populations than the latter were among themselves, indicating sufficient representation of wild populations in zoo populations. The patterns we uncover support continued collaboration of ex situ and in situ endeavours as supplementation will likely be required for the long-term viability of the very wild populations the zoos rely on for genetic sustainability.

## Introduction

Human actions have placed more species at risk of extinction now than ever before^1^, with more and more species requiring active management both in situ (hereafter “wild”) and ex situ (ex. zoos and aquaria; hereafter “zoos”). Yet, while conservation science has often prioritized in situ efforts, deeming methods such as captive breeding a last resort^2^, the role of zoos in conservation has become increasingly more important and more accepted^3^. Zoos provide a unique setting for research and conservation of wildlife, hosting captive populations both as an assurance against extinction and for the purpose of conservation breeding. The latter programs strive to breed offspring ex situ for release in situ*,* thus boosting wild census population sizes and standing genetic diversity^4,5^ and providing time for threats in the species’ native habitat to be reduced or mitigated. As in situ and ex situ efforts complement and rely upon one another, they should be planned and managed collaboratively^6,7^.

### Genetics in zoological conservation

While an explicit goal of breeding and reintroduction programs is the conservation of genetic diversity, there are inherent genetic risks associated with breeding small groups of animals ex situ and releasing them into the wild. Populations held ex situ may become adapted to captivity^8,9^, experience reductions in effective population size through increased variance in family size^10^, and face concomitant increased risks of inbreeding depression and loss of genetic variation^11,12^. The release of zoo-sourced individuals might then depress rather than improve the mean fitness of the population(s) they supplement and reduce the probability of a species’ long-term persistence^5^. Therefore, a critical question for all conservation breeding programs is whether they will improve, or at least maintain, the genetic variation of wild populations. To this end, zoo populations must be demographically robust and genetically representative of the wild populations they are derived from and both must be managed for long term sustainability^13^. It is generally accepted that ex situ populations should maintain 90% of wild genetic diversity over 100 years and minimize inbreeding where possible through the use of pedigrees or parentage tracking^14,15^. Inbreeding can be tracked using population metrics such as expected and observed heterozygosity, and inbreeding coefficients^16^. While expected heterozygosity (He) measures allelic diversity at the population level, observed heterozygosity (Ho) measures it within individuals, making He a more informative metric for comparing genetic diversity across ex situ and in situ populations. Wright’s F_IS_ statistic directly assesses discrepancies between He and Ho as they indicate deviations from Hardy–Weinberg equilibrium^17^.

Intentional management of breeding populations can also bring ex situ programs closer to a sustainable effective population size^13^ (generally, Ne of at least 500). Ne measures the rate of change (particularly loss) of genetic diversity due to drift across generations^18,19^. As opposed to the current state of genetic diversity, Ne informs inferences on future genetic diversity changes and is highly suited for monitoring conservation actions^20^. Monitoring genetic metrics such as genetic diversity (He and Ho), inbreeding coefficients (F_IS_), and effective population size (Ne) is critical to guide management of captive populations towards long-term ex situ persistence and offspring suitable for supplementation or reintroduction efforts^13^. Indeed, a recent study found that the genetic diversity retained in translocation efforts for small populations could be significantly improved by optimizing the composition of individuals^21^. Such optimization not only requires basic genotyping of source populations but is dependent on the genetic variation available within, as well as the level of genetic differentiation between, populations^21^. Understanding the genetic variation available in all wild populations is also crucial in identifying sustainable source populations and ensuring these genetic lineages are maintained ex situ if their survival in the wild is at risk^22^. Thus, assessing genetic variation in both zoo and wild populations is essential for informed and effective conservation breeding and reintroduction programs.

### Amphibian extinction crisis

Globally, amphibians are more at risk of extinction than reptiles, birds, or mammals, facing a suite of threats including habitat loss, invasive species, and several infectious diseases^23,24^. Amphibian species are also particularly prone to loss of genetic diversity^25^; they often have naturally low effective population sizes^26–28^ which, coupled with their overall population declines, leaves them more vulnerable to genetic drift and inbreeding^25^. Habitat fragmentation, one of the most severe threats to amphibians, creates smaller sub-populations with reduced gene flow, thereby increasing genetic differentiation between them while genetic diversity within them erodes much faster^29,30^. Even in unfragmented habitat, local extinctions are common^31^ and naturally low dispersal rates (common to many amphibians) increase the risks of genetic drift^25^.

In the face of these threats, amphibians are prime candidates for ex situ breeding, due to their relatively low holding costs and high reproductive output, leading to high conservation impact^32,33^. The number of amphibian collections in zoos has particularly increased in response to chytridiomycosis, a deadly disease devastating wild populations of amphibians worldwide^3,34,35^. Yet, tracking ex situ amphibian pedigrees, necessary for genetic management, has proven challenging because they are often managed in large groups, breed communally, and show high inter-individual variation in reproductive output, making the retention of genetic lineages difficult^36^. One highly vulnerable species under ex situ management is the Oregon Spotted Frog (*Rana pretiosa*), Canada’s most endangered anuran^37^.

### The Oregon Spotted Frog

The Oregon Spotted Frog had already been lost from as much as 90% of its extrapolated historical range by 1997^38,40^ and the 6 remaining Canadian populations are estimated to have fewer than 250 breeding pairs each^41^. The continuous loss of suitable wetlands, human intrusion and disturbance, invasive species, and pollution are only a few of the threats faced by this species across its limited range within British Columbia^37,39^. Moreover, habitat fragmentation has left all extant populations genetically isolated and susceptible to inbreeding depression, further loss of genetic diversity, and to stochastic events^39,41^. Due to their highly threatened status, *R. pretiosa* thus require ex situ conservation in addition to ongoing mitigation of in situ threats^3^.

Two zoos and one aquarium (hereafter “zoos”) established independent but coordinated conservation breeding and reintroduction programs for *R. pretiosa* in the 2000’s, but the genetic sustainability of these ex situ populations has not been assessed. The limited work on *R. pretiosa* genetics focused on populations from its American range, finding low genetic diversity in the wild^37,42,43^. Data collected in 2009 suggested that Canadian populations were distinct from American ones, displayed small effective population sizes, and were likely experiencing inbreeding (Blouin pers. comm. 2009 in COSEWIC 2011). Since collecting individuals from already genetically impoverished populations into captivity can depress population sizes, increasing the risk of inbreeding and genetic drift^44^, it is imperative to assess the current level of genetic diversity in both zoo and wild *R. pretiosa* populations.

Using Genotyping-By-Sequencing (GBS), we investigated patterns of genetic variation and structure in both ex situ and in situ populations with the goals of (i) assessing whether this measured variation might be sufficient for long term genetic sustainability of the species in Canada and (ii) informing reintroduction efforts based on current population structure. We compared genetic diversity between and among three zoo and five wild *R. pretiosa* populations, predicting that it could be either lower in zoos due to founding effects or higher due to cross-breeding of differentiated wild sources. We also estimated the effective population sizes (Ne) of both wild and zoo populations and calculated their inbreeding coefficients (F_IS_). We predicted zoos would have relatively high Ne (compared to census size N) due to management of population demographics, but higher F_IS_ than wild populations due to fewer mating options between non-related individuals in the zoos. Finally, we assessed wild *R. pretiosa* population structure to inform ex situ demographics and breeding strategies, predicting each population would be highly differentiated due to their isolated locations, limited dispersion capabilities, and small Ne-induced drift.

## Results

### Genetic diversity

**Fig. 1:**
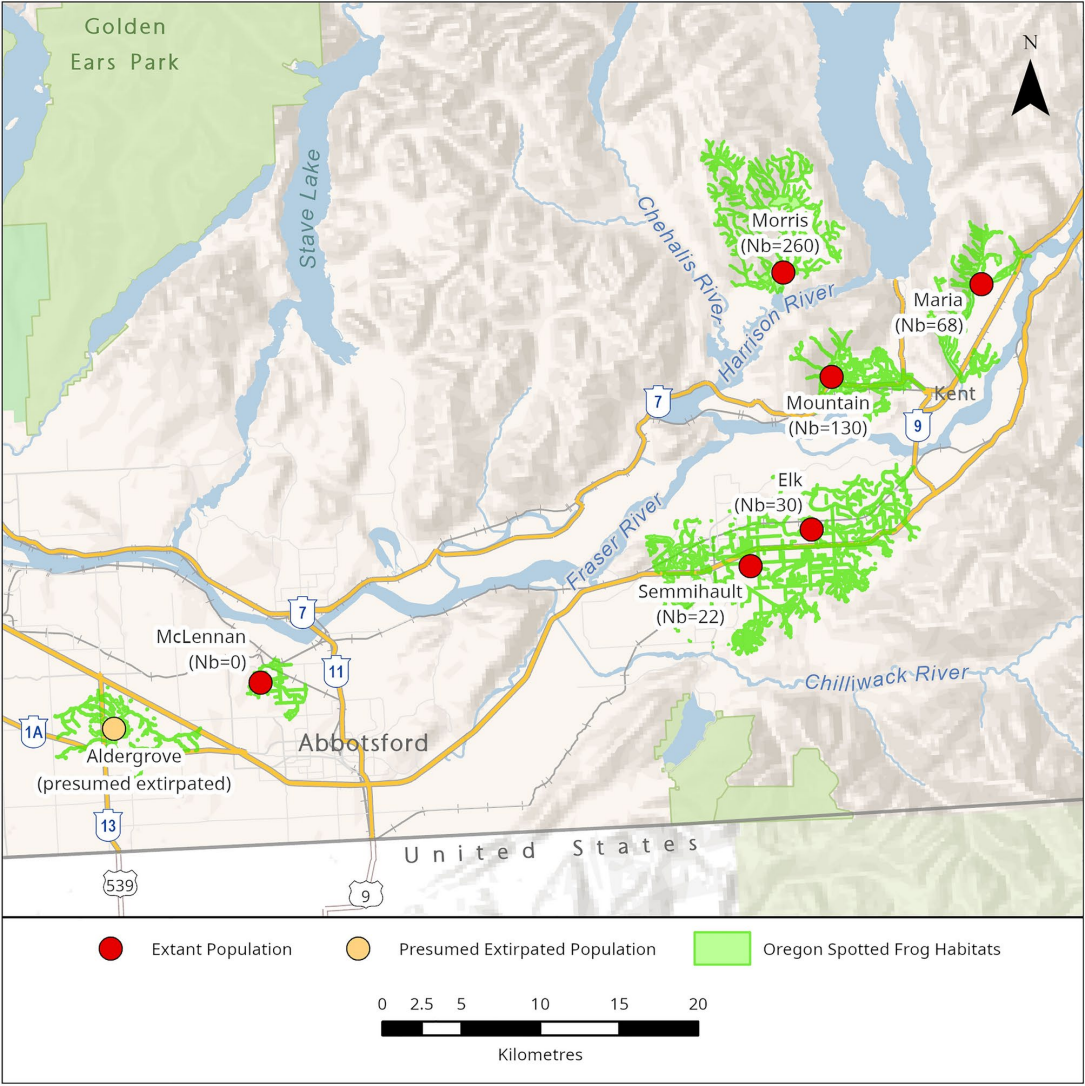
The remaining wild populations of Oregon Spotted Frog in British Columbia, Canada. Each extant population is indicated by a red circle within the known watershed (coloured green), with the estimated breeding population size (Nb) from egg surveys in 2022. McLennan is still considered extant but no egg masses or frogs have been seen since 2017. Aldergrove is presumed extirpated (orange circle) as no *R. pretiosa* have been found there since 2006. Map produced in ArcPro 3.2.2 in NAD 1983 BC Environmental Albers.

**Fig. 2:**
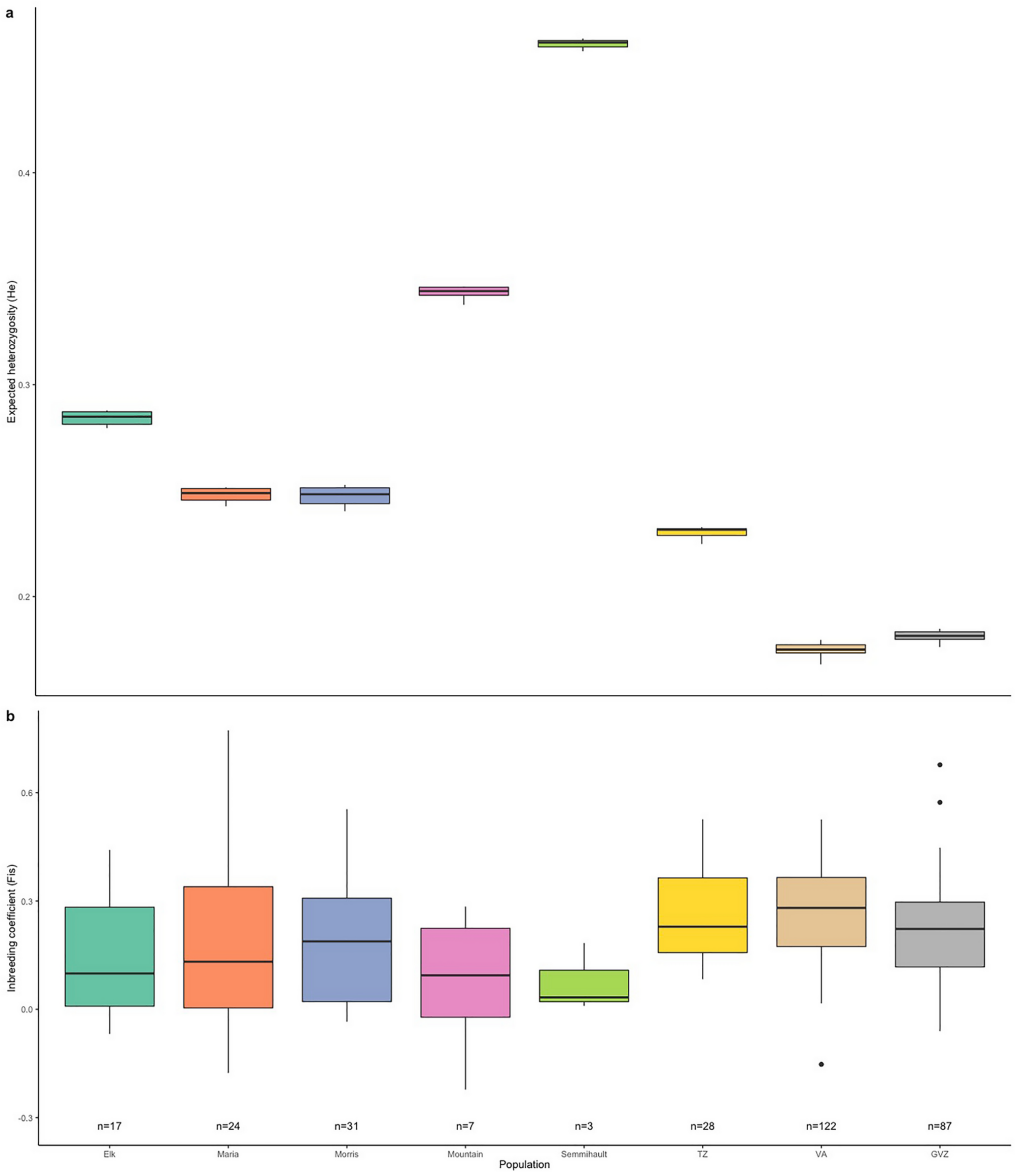
Genetic diversity estimates for all Oregon Spotted Frog populations sampled in Canada. Mean estimates of **a)** expected heterozygosity (He), and** b)** inbreeding coefficients (F_IS_) at five wild (Elk, Maria, Morris, Mountain, Semmihault) and three zoo (TZ, VA, GVZ) populations.

Based on total DNA extracted from 322 individuals we constructed a GBS library (using *Pstl/Mspl restriction enzymes)*, performed Illumina Novaseq sequencing, filtered the raw data and called single nucleotide polymorphisms (SNPs) with STACKS v.2.62^45^ (*denovo* mode). This resulted in a total of 22,230 SNPs genotyped across 321 samples from three zoo and five wild populations (Fig. 1). The mean (across called SNPs) expected heterozygosity across wild populations ranged from 0.248 (Morris) to 0.461 (Semmihault) and across zoo populations from 0.175 (VA) to 0.230 (TZ) (Table 1). When samples are pooled, mean He in zoos (n = 239) was significantly lower than in the wild (n = 82; t-test, 88.91 df, p < 0.05). Pairwise comparisons of both zoo and wild populations identified GVZ as having significantly lower He than every population except Semmihault (Dunn’s test p = 0.064; Fig. 2a). Note, this lack of significance may have resulted from lower power, with Semmihault’s small sample size (n = 3). The He for VA was significantly lower than every other population, both wild and zoo (Dunn’s test p < 0.05). Observed heterozygosity was consistently lower than expected heterozygosity for all populations (Table 1).

**Table 1:** Mean genetic diversity indices for Oregon Spotted Frog populations calculated from a total of 22,230 SNPs. The first two rows display average values for combined zoo (from populations TZ, VA, GVZ) and wild (from populations Elk, Maria, Morris, Mountain, Semmihault) individuals respectively. N = sample size, Ho = observed heterozygosity, He = expected heterozygosity, F_IS_ = inbreeding coefficient, Ne = effective population size. The number of breeding adults (Nb) was determined by egg mass surveys for wild populations and represented the total number of adults for zoo populations.

POPULATION	N	H_o_	H_e_	F_IS_	N_e_	N_b_
**ZOOS (n = 3)**	239	0.139	0.184	0.248		
**WILD (n = 5)**	82	0.230	0.271	0.158		
Elk	17	0.242	0.284	0.147	12.5 (7.6—22.3)	112
Maria	24	0.208	0.248	0.165	4.4 (2.5—8.5)	154
Morris	31	0.202	0.248	0.185	19 (12.1—32.1)	348
Mountain	7	0.317	0.343	0.080	*	102
Semmihault	3	0.427	0.461	0.075	*	22
TZ	28	0.170	0.230	0.263	16.1 (11.4 - 23.9)	19
VA	122	0.129	0.175	0.265	34.8 (29.4 - 41.5)	111
GVZ	87	0.142	0.181	0.220	31.8 (26.7 - 38.2)	98

*Values could not be computed accurately due to low sample size.

Inbreeding coefficients (F_IS_) ranged from 0.075 (Semmihault) to 0.265 (VA; Table 1). Elk’s F_IS_ was significantly lower than VA (Kruskal–Wallis, p = 5.535e-05) but the post-hoc Dunn’s test was only nearly significant (p = 0.056; Fig. 2b). Zoo and wild category mean inbreeding coefficients differed significantly (t-test, 107.77 df, p = 0.0001) with mean values of 0.248 and 0.158, respectively. Effective population sizes (Ne) for populations with at least 15 individuals ranged from 4.4 (Maria) to 19 (Morris) in the wild (Ne was not calculated for Mountain and Semmihault due to low statistical power), and were 16.1, 31.8, and 34.8 for TZ, GVZ, and VA, respectively (Table 1). Not unexpectedly, the breeding population sizes (Nb) estimated from egg mass surveys were much larger than the estimated Ne values (Table 1).

**Fig. 3:**
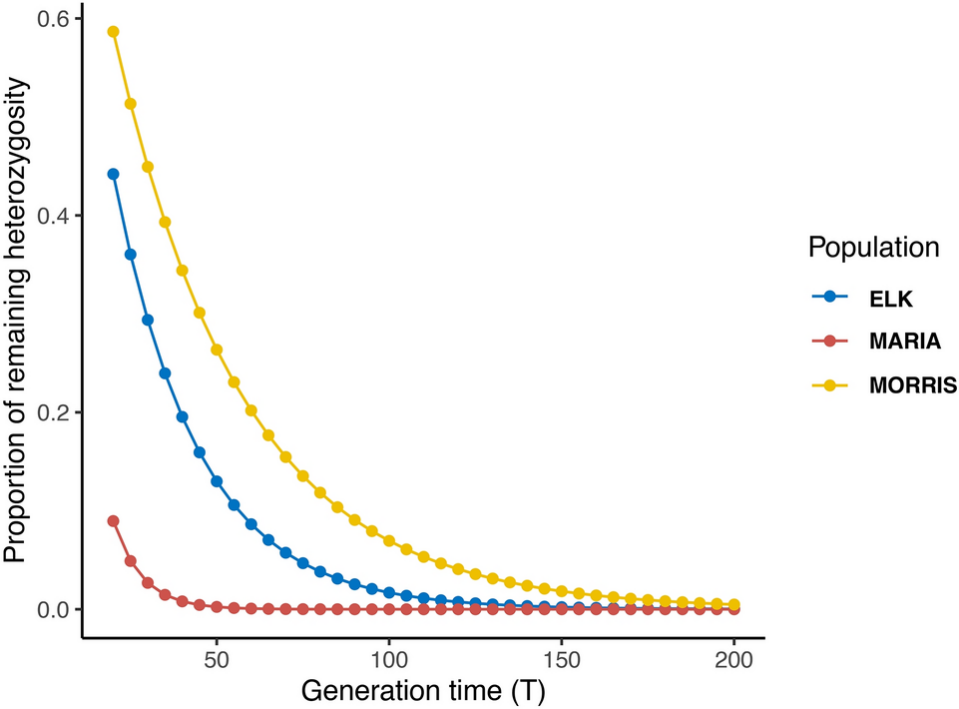
Heterozygosity loss through time in three wild populations of Oregon Spotted Frog. The proportion of remaining heterozygosity was inferred from effective population size estimates (Ne) for T = 20 to T = 200. Generation time (T) for *R. pretiosa* is 2 years.

Using estimated Ne values, we projected the loss of heterozygosity over time and observed that Maria, with a mean Ne of 4.4, starts with a lower proportion of remaining heterozygosity and consequently is expected to lose all genetic diversity within 50 generations (one generation = 2 years). Elk and Morris, which start with a higher proportion of heterozygosity, are projected to take 150 and 200 generations for complete loss of genetic diversity respectively (Fig. 3).

### Population structure

**Fig. 4:**
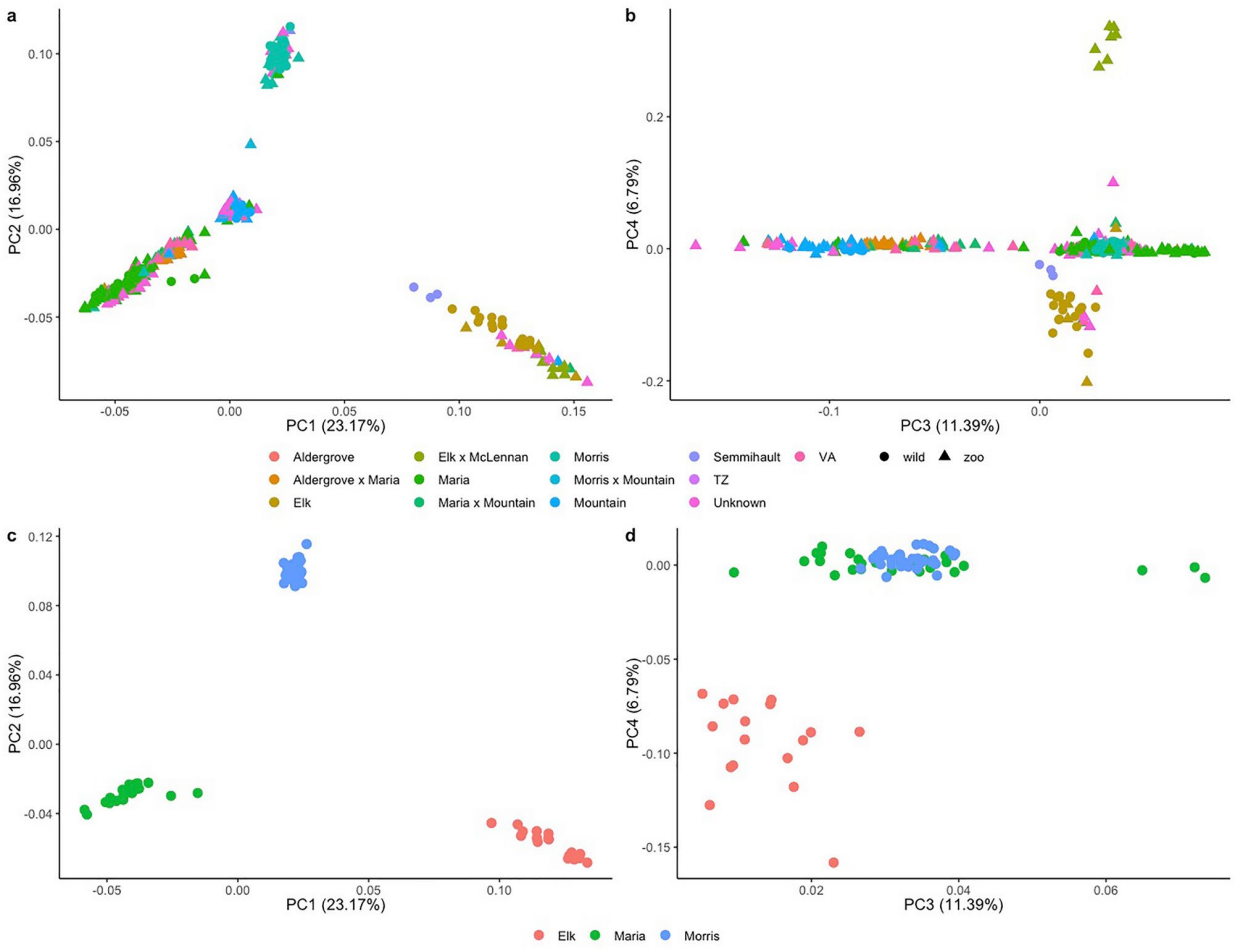
Principal component analyses of Oregon Spotted Frogs based on SNP genotypes. Each point is an individual, coloured by source population. Each axis indicates the amount of variation respectively explained in the data. **a**) and **b**) contain all sampled frogs coloured by their genetic source. Wild-caught frogs are indicated by circles and frogs sampled in zoos are indicated by triangles. Cross-bred zoo frogs are indicated by an ‘x’ in the name (e.g. Aldergrove x Maria Slough). Zoo frogs with unknown parentage are indicated as “unknown” or according to their respective zoo (if many-generations zoo-born). **c**) and **d**) contain only the three wild populations with ≥ 15 samples.

A principal component analysis (PCA) conducted on genotypes of all individuals revealed an absence of clustering according to zoo *vs.* wild populations but rather exhibited clustering according to individual genetic source, with zoo frogs clustering according to their respective genetic sources (*i.e.* their tracked lineage). Three main clusters, with a less distinct fourth, were differentiated by the first and second PC axes (PC1: 23.17%, PC2: 16.96%; Fig. 4a). Among the wild populations, Elk and Semmihault clustered together, while Maria, Morris, and Mountain form unique clusters, with Mountain and Maria in close proximity (Fig. 4a). The third PC axis (11.39%) differentiated a Mountain cluster from a cluster consisting of Maria + Morris, while the fourth PC axis (6.79%) delineated a cluster of Elk + Semmihault from zoo cross-breeds of Elk x McLennan (Fig. 4b). A PCA ordination using just the three largest wild populations (Elk, Maria, and Morris) differentiated each on the first and second PC axes (Fig. 4c), while the third and fourth PC axes differentiated a Maria + Morris cluster from Elk (Fig. 4d).

**Fig. 5:**
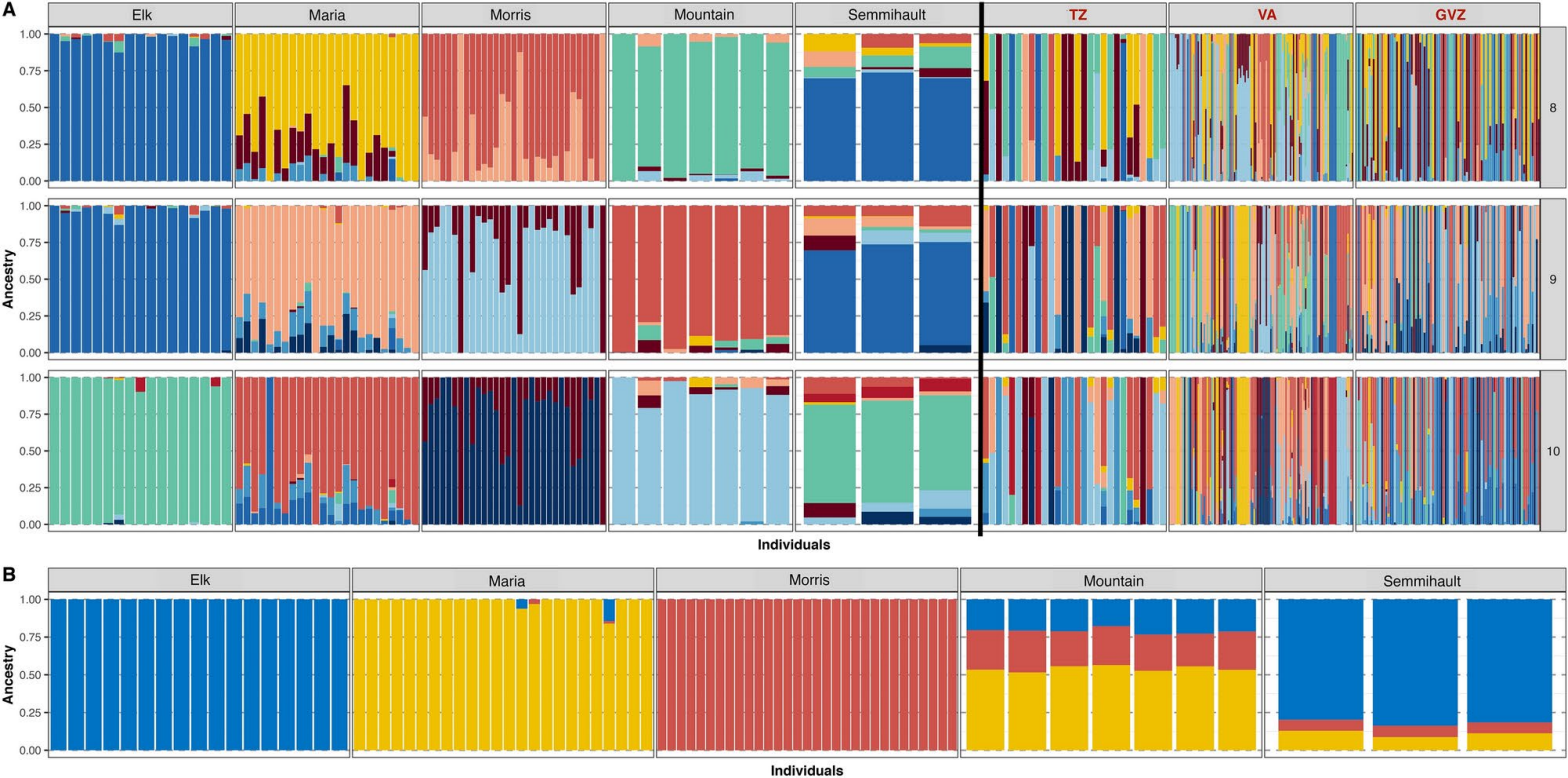
ADMIXTURE population structure analysis with individuals grouped by population. **A**) Wild populations (Elk, Maria, Morris, Mountain, Semmihault) on the left in black text, and zoo populations (TZ, VA, GVZ) on the right in red text, displayed for K = 8 to K = 10 where K is the inferred number of ancestral populations. **B**) Wild populations only, for K = 3.

We also investigated genetic structure in ADMIXTURE for K ranging from 2 to 15. Cross-validation indices (Supplementary Material, Fig. S1) and population history suggested K = 8–10 (Fig. 5a). When grouping ADMIXTURE results by population, wild populations formed distinct clusters in comparison to the very mixed zoo populations. Elk formed a distinct cluster at K = 8–10 (Fig. 5a) and remained distinct all through K = 15 (Supplementary Material, Fig. S2). Morris was a distinct cluster until K = 7 but became mixed at K = 8, sharing some of its ancestry here with Mountain; for K = 9–10 however, Morris was still mixed but no longer clustering with Mountain (Fig. 5a). Mountain remained a relatively distinct cluster until K = 13, with the aforementioned grouping with Morris occurring at K = 8 only. Maria showed more variation than the other wild populations and had 24% mixed ancestry by K = 5 (Supplementary Material Fig. S2). Zoo populations were all highly mixed from K = 2 onwards, displaying all the wild population ancestry and showing some unique ancestry as well (Fig. 5a). When grouping individuals by genetic source, zoo cross-breeds (Elk x McLennan) clustered with Elk until K = 10, after which they formed a distinct cluster (Supplementary Material, Fig. S3). Aldergrove x Maria did not cluster with Maria but showed distinct ancestry through K = 8–10 aligning primarily with Maria x Mountain crosses for K = 8 but forming its own distinct cluster for K = 9–10. Individuals with Elk in their genetic source presented distinct (≥ 95% unmixed) bars. Since Maria itself was mixed when K = 8–10, its main ancestral sources (indicated by different colours in Fig. 5a) were all present in zoo populations, appearing there as both mixed and distinct bands. Where Morris appeared in zoo populations, it almost always formed distinct bands. When analysing the five wild populations alone, the best statistical K was 3 (Supplemental Material, Fig. S4), with Elk, Maria, and Morris representing distinct clusters and Mountain and Semmihault being mixtures of the other three (Fig. 5b). The Semmihault individuals grouped primarily (82%) with Elk while the Mountain bands grouped primarily (54%) with Maria. For K = 4 however, Mountain formed a distinct cluster while Semmihault remained grouped with Elk (Supplemental Material, Fig. S4).

Weir and Cockerham’s Fst-values were calculated for the zoo and wild categories as well as for individual populations. Comparing the combined zoo population samples and the combined wild population samples resulted in Fst = 0.012 (ranging from 0 to 0.232 across genomic regions). All zoo populations were < 1% differentiated from one another (Table 2) while the average pairwise Fst between wild populations was 0.105, ranging from 0.088 (between Maria and Morris) to 0.116 (between Elk and Morris; Table 2). Pairwise Fst between individual wild and zoo populations were lower than between wild populations themselves (Table 2), with Elk the most differentiated from all zoo populations (mean Fst [0.068–0.082]) and Maria the least differentiated (mean Fst [0.012–0.024]). Furthermore, while average Fst values remained below 0.12 among all three wild populations, some genomic regions were highly differentiated (e.g. 0.975, 0.979, and 0.963 maximum range values; Table 2).

**Table 2:** Mean pairwise Fst estimates (range in brackets) across Oregon Spotted Frog SNPs. Zoo populations are: Vancouver Aquarium (VA), Greater Vancouver Zoo (GVZ), and Toronto Zoo (TZ). Wild populations are: Elk, Maria, and Morris. Two wild populations were not included due to low sample size (Semmihault and Mountain).

**Population**	VA	GVZ	TZ	Elk	Maria
GVZ	0.01 [0; 0.29]				
TZ	0.003 [0; 0.28]	0.008 [0; 0.32]			
Elk	0.068 [0; 0.835]	0.082 [0; 0.84]	0.068 [0; 0.826]		
Maria	0.022 [0; 0.499]	0.012 [0; 0.504]	0.024 [0; 0.568]	0.111 [0; 0.975]	
Morris	0.041 [0; 0.729]	0.053 [0; 0.70]	0.053 [0; 0.812]	0.116 [0; 0.979]	0.088 [0; 0.963]

## Discussion

Conservation breeding programs are often established to “buy time” for species on the brink of extinction^6^. Such programs have played a critical role in reducing the threat level in 16 of the 64 species down-listed by the IUCN^46^. In amphibians, the number of species managed ex situ for conservation breeding and reintroduction has greatly increased since 2007^34,47^ but not all of these species are in fact suitable for ex situ actions^48^. Endangered species with existing conservation breeding programs should be assessed to ensure ex situ actions are warranted^48^ as successful breeding ex situ is not sufficient in itself^36^ but offspring must have sufficient genetic diversity for short and long-term population adaptation to the wild as well^13^. Our study of Oregon Spotted Frogs in Canada underscores the importance of monitoring genetic variation in both ex situ and in situ populations to assess the efficacy of genetic management strategies ex situ and the use of genetic sources in recovery programs^49,50^. This and our analysis of population structure indices provide a basis for practical recommendations for conservation breeding and reintroduction efforts more broadly.

The genetic diversity of American populations of *R. pretiosa* is low compared to other ranids^37,42,43^, and here the three zoos hosting breeding populations of *R. pretiosa* display even lower genetic diversity than the few wild populations remaining in Canada. These zoos have retained only 68% of wild genetic diversity measured as He (0.184 compared to 0.271), falling below the 90% retention target for sustainable ex situ populations. Interestingly, however, both VA and GVZ had similarly low He despite divergent conservation breeding strategies. VA tracks parentage and assigns small breeding groups (n = 2–6) in attempts to reduce mean kinship and maximize genetic diversity. On the other hand, GVZ takes a communal breeding approach, separating *R. pretiosa* into three large tanks at random (n = 30), allowing more natural mate selection but making it impossible to track or manage genetic pedigrees. TZ follows a similar genetic management strategy to VA but hosts a very small population of *R. pretiosa* (n = 20 on average) which inherently limits mating options. Despite these varying setups and strategies, VA hosts the lowest genetic diversity, followed by GVZ, then TZ, and then the wild populations. This suggests that a communal breeding setup, allowing for more natural mate selection, may not only have reproductive and behavioural benefits^51^ but may also maintain genetic diversity and mitigate inbreeding in this species.

Inbreeding coefficients (F_IS_) did not vary as significantly as heterozygosity at the population level but the zoos as a category still had significantly higher F_IS_ than the wild category. All populations, whether zoo or wild, had a positive F_IS_, highlighting the mean deficiency of heterozygotes among individuals with respect to that expected across populations (seen in the consistently low Ho compared to He in Table 1). A positive F_IS_ can indicate mating between close relatives, assortative mating, and/or the existence of a Wahlund effect. Limited mate availability can mimic assortative mating patterns^52^ but currently, the zoos with the highest Ho:He discrepancy (VA and GVZ) have estimated population sizes similar to those seen in the wild, suggesting mates are no more limited at the population scale in zoos than in the wild. Further, the use of pedigrees to track and manage mate pairings at VA should reduce the possibility of assortative mating yet VA has the highest F_IS_ value (0.265). With that said, VA historically managed its pedigrees to minimize known relatedness in breeding groups while simultaneously trying to keep certain lineages distinct (*i.e.* breeding Aldergrove x Aldergrove descendants). It is possible that managing for these specific lineages increased the opportunity for assortative mating in comparison to the less-controlled, communal breeding at GVZ. Given these results, VA should reassess its pedigree strategy and/or move to a more communal breeding setup if possible.

Inbreeding, however, may not severely impact *R. pretiosa*. Other vulnerable species have been found to persist with high inbreeding levels^53^; regardless, all *R. pretiosa* populations should be monitored for signs of inbreeding depression (e.g. decreasing fertility and offspring survivorship). It is accepted that founding zoo populations with fewer than 200 individuals increases susceptibility to genetic drift, inbreeding depression, and demographic stochasticity, and leads to decreases in effective population size and genetic diversity^15,54^. While VA, GVZ and TZ are exhibiting some of these declines, other zoos have been able to maintain stable genetic diversity and inbreeding levels despite few founders and small population sizes^55,56^. Many zoo populations rely on cooperative management to effectively prevent demographic and genetic declines and Che-Castaldo et al.^55^ argue that such stability is an achievement in itself under the current biodiversity crisis. Relatively low Ne has been observed in amphibian populations in the wild^26–28^, as Ne is affected by many factors including variance in reproductive success^57^ and limited dispersal ability^58^. Through careful management of these factors, such as maximizing the number of breeding individuals, stabilizing population sizes across generations, and equalizing operational sex ratios ex situ, zoos can increase their Ne^13^. Such effective management is taking place at all three zoos where we estimated higher Ne-values relative to wild populations despite the latter harbouring more than twice the number of breeding adults (Nb) according to ongoing population monitoring. While Ne in all three zoo populations remains well below the conservative goal for short term persistence (Ne = 50^59^) and the threshold to avoid inbreeding depression (Ne ≥ 100^60^), increasing Ne in zoos relative to the wild will help improve retention of genetic diversity.

While used by the World Association of Zoos and Aquariums^61^, it is worth noting that the standards by which we here assess the ‘genetic condition’ of *R. pretiosa* are derived primarily from studies on mammalian species^13–15^. In their general guidelines for amphibian captive breeding programs, the Amphibian Ark^62^ recommends founding populations with a minimum of twenty breeding pairs (i.e. an Ne = 40), which falls far below other Ne standards based primarily on mammals^13–15,59,60^, perhaps reflecting the low Ne observed in many wild amphibians^26–28^. For zoos to accurately measure and manage the genetics of ex situ populations, a practice critical to ensuring population sustainability^13^, we need standards relevant to species with diverse life histories. Many amphibians do not fit easily into typical methods of genetic management (*ex*. mean kinship) due to the larger numbers of individuals housed and their different reproductive modes^63^. In response to this challenge, the Amphibian Ark has developed more pertinent standards of genetic management^63^, but ultimately the ability to compare zoo and wild genetic metrics may provide the most accurate proxy of genetic condition due to species-specific characteristics.

The Oregon Spotted Frog may have had low Ne and genetic diversity in the wild for many generations. Rapid reduction in genetic diversity due to anthropogenic effects (*i.e.* habitat fragmentation) enhances the risk of inbreeding depression^64^, but if a species has existed for generations with low genetic diversity, many deleterious alleles may have already been purged, decreasing the risk of future inbreeding depression^25^. Although *R. pretiosa* have experienced continued habitat fragmentation over the last 150 years^37^, it is possible that subsequent sub-populations persisted in their fragmented landscape with low genetic diversity. Cushman^31^ has suggested that species with low dispersal rates have higher-than-expected survivorship in small and fragmented habitats due to decreased mortality risks associated with dispersal. For example, *R. pretiosa* do not experience the road mortality rates of other amphibians^65^, perhaps contributing to their persistence. Future studies should also assess allelic diversity as other studies have found allelic diversity to decrease more rapidly than heterozygosity^5,66^. Kraaijeveld-Smit et al.^67^ found allelic diversity decreased in Mallorcan toad breeding programs after 3–8 generations while heterozygosity took 12 generations to decrease in comparison to wild populations. This discrepancy is not unexpected, since allelic richness is more sensitive than heterozygosity to bottlenecks^67^. Allelic richness should therefore be considered in conservation genetics to inform breeding strategies and recovery actions, particularly in light of potential local selection.

Our genetic structure analysis revealed that average Fst values were moderate^68^ among wild populations of *R. pretiosa* (Fst [0.09–0.12]) and low among zoos (Fst [0.003–0.01]) and between zoos *vs*. wild populations (Fst [0.012–0.082]). In particular, biologically relevant differentiation was observed between Elk and Maria, and Elk and Morris, with mean pairwise Fst > 0.1. In their study on *R. pretiosa* genetics, Blouin et al.^42^ sampled three Canadian populations and found they were all well differentiated from one another but suggested this finding was inflated by small sample sizes. Yet, while average Fst values are only moderate among wild populations, some genomic regions were highly differentiated (maximum Fst = [0.96–0.98]). Further research should be conducted (ideally with the aid of a reference genome) to investigate whether these regions of near fixation may be due to local adaptation *vs.* random genetic drift, as is more frequent in isolated populations^69,70^. For example, several studies have reported small, isolated populations of amphibians in fragmented landscapes, especially species with low dispersal capability and high site fidelity^71,72^, to be highly differentiated^29,30^. In this context, the low to moderate average Fst values reported here are noteworthy. Rivers have mixed effects on amphibian dispersal^73,74^ and the Fraser River may have historically improved gene flow between our populations while extensive dyking systems built to prevent flooding may have reduced recent gene flow^39^. Both the ADMIXTURE and PCA results also indicate historical admixture of populations in the Fraser River floodplain (Maria, Morris, Mountain) consistent with *R. pretiosa* being known to travel almost exclusively by water^65,75^. While no highly differentiated genomic regions appeared in Fst comparisons among zoo populations, it would be pertinent to study the survivability of zoo-bred frogs in the wild to determine whether particular ancestries (*ex.* Elk *vs.* Maria) have higher survival rates than others. Currently, all zoo-bred frogs are released at one reintroduction site within the Maria Slough watershed, but the potential for local adaptation should be considered before reintroducing zoo-bred frogs into other watersheds.

Maria shows the most admixture among the extant wild populations, and this admixture is also visible in all zoo frogs with Maria as their genetic source. The lack of clustering with other extant populations, however, suggests Maria was once connected to ancestral sources, perhaps from further upriver. The low genetic diversity and high inbreeding observed in Maria may indicate these sources, if still extant, are no longer connected to Maria^76^. Interestingly, some of these ancestral genetics have been carried into the zoo populations, evidenced by zoo frogs with distinct ADMIXTURE ancestry (*i.e.* unique colours) but Maria parentage. More recently extirpated populations (*e.g.* Aldergrove) are also represented in zoo populations, which explains why the optimal K for all populations (zoo and wild; K = 8–10) is more than 2 × larger than that estimated for only the five wild populations (K = 3). It will be critical to identify these unique individuals and ensure persistence of these ancestral lines in the breeding programs to carry on this impressive level of genetic representation. Of the remaining extant *R. pretiosa* populations, Elk and Semmihault show a separate and perhaps ongoing connectivity. These two populations lie in a watershed network of ditches but known frog occurrences are separated by more than 3 km, their maximum travel distance^75^. Importantly, the hydraulic connectivity of the ditch networks is currently not well understood. These ditches generally lack the stability of shallow shelf oviposition habitat which occurs more readily in slough and wetland habitat types^39^, and may be prone to more frequent, high velocity flows. It is possible the continuous corridor of deep water in this ditch habitat increases movement and connectivity as larger *R. pretiosa* movements (> 1 km) have been documented along analogous linear riparian systems in the past^75^. Human-modified environments can sometimes unintentionally increase connectivity or create additional habitat for amphibians^70^ and it is thus possible that this human-made ditch system has increased connectivity between Elk and Semmihault. Such a connection should be thoroughly investigated and protected given the role these populations might play in the long-term sustainability of *R. pretiosa* in Canada.

While the zoo populations assessed here exhibit declines in genetic diversity and increased inbreeding coefficients, the state of wild *R. pretiosa* populations in Canada is so dire that conservation breeding efforts may yet prove essential to the recovery of the species. One of the primary source populations for the conservation breeding programs (Maria) is projected to lose all genetic diversity within 50 generations. This not only highlights the necessity of conservation breeding and reintroduction programs to ensure the persistence of *R. pretiosa* genetic diversity, but also suggests some changes should be made to their implementation. The frequent collections from Maria may have contributed to its genetic decline, in turn jeopardizing the sustainability of the zoo populations which depend on both their internal population genetics and that of their source populations^13^. Thus, external supplementation of zoos should be adjusted both to protect Maria and to diversify zoo population demographics so as not to rely too heavily on one source. Optimizing the individual composition of zoo populations can also significantly improve the genetic diversity they harbour^21^. Together, this suggests that incorporation of more Mountain, Elk, and Semmihault frogs into zoo populations should increase ex situ genetic diversity as these populations have the highest genetic variation of all wild populations.

Due to capacity constraints, increasing the overall size of zoo populations may not be feasible, therefore zoos must maximize the positive impact of the limited numbers of individuals they can hold. Optimization of zoo genetics also depends on the level of differentiation among sources^21^ so all offspring should be monitored for signs of both inbreeding and outbreeding depression. While concerns for the latter are increasingly hailed as overstated, it may take generations to manifest^77^, so monitoring is warranted. Such monitoring will also inform direct supplementation of the Maria population, which should be strongly considered lest we monitor this population to the point of extirpation rather than risk taking in situ action alongside ongoing ex situ actions^78^. The collaboration of both ex situ and in situ partners in the *R. pretiosa* recovery team has already contributed much to the persistence of the Oregon Spotted Frog in Canada through the establishment of a viable reintroduction population and this study suggests continued, unified efforts between these two arms of conservation may prove an essential strategy for the genetic sustainability of endangered species in general.

## Methods/analysis

### Sampling

All extant wild populations of *R. pretiosa* in Canada – Morris Valley (Morris hereafter), Mountain Slough (Mountain hereafter), Maria Slough (Maria hereafter), Elk Brook (Elk hereafter), Semmihault Creek (Semmihault hereafter), and McLennan – were sampled in the spring of 2021. These populations are between 3 and 55 kms apart, occurring in floodplain marshes, sloughs, or channelized watercourses in the Fraser River Lowlands of British Columbia with low to moderate amounts of emergent vegetation and silty substrate^39^ (Fig. 1). Egg mass surveys are conducted annually in the spring (March–May) at each of these sites and a more extensive capture-mark-recapture program is ongoing at both Maria and Morris. The Aldergrove population is presumed extirpated but remains represented ex situ by a few frogs of Aldergrove descent.

Two zoos and one aquarium currently run conservation breeding and reintroduction programs as part of a combined recovery effort for this endangered species. Due to differing facilities, resources, and location, each zoo has a unique program and approach. The Vancouver Aquarium (VA) began its breeding program in 2010 and regularly holds 100–200 *R. pretiosa*. The Toronto Zoo’s (TZ) program, established as both a breeding program and assurance population, started in 2010 as well and holds an average of 20 *R. pretiosa* at a time. While the Greater Vancouver Zoo (GVZ) has helped headstart *R. pretiosa* since 2003, their year-long breeding program did not begin until 2017 but now holds roughly 80–100 *R. pretiosa*. The initial founding populations of all three zoos were composed of *R. pretiosa* collected primarily from Maria and Morris, with some from Mountain and Aldergrove (before its presumed extirpation). Elk and Semmihault were not discovered as populations until 2015 but were subsequently incorporated into the zoo populations. The VA and GVZ populations are frequently supplemented with eggs collected from any or all extant wild populations under the general goal of retaining reproductive lines from all extant Canadian populations. TZ is supplemented less frequently due to capacity constraints and typically receives frogs at least one-year-old from either VA or GVZ.

Genetic samples were collected primarily by buccal swabbing^79^, an effective and less harmful alternative to toe clipping. Each swab was collected by gently prying open the frog’s mouth with a flat tip (*i.e.* guitar pick) and rolling a sterile cotton swab around the inside of the mouth^80^. Swabs were dried and stored at −20 °C until DNA extraction. When dead *R. pretiosa* were found in the wild or captivity, tissue samples were collected (approximately 5 × 5 mm sample of skin or muscle) and stored in ethanol at −20 °C. A total of 7–30 unique swabs were collected from each of the four wild populations (Maria, Morris, Mountain, Elk) during the breeding season (March–April), when frogs were most active. In addition, five eggs each from different egg masses were collected and stored in ethanol at −20 °C for the wild populations Elk and Semmihault, as no frogs were captured at the latter site. No eggs or frogs were found at McLennan in 2021 but this lineage remains represented in zoo frogs. Buccal swabs were also collected from all mature (≥ 2 years old) *R. pretiosa* at the Vancouver Aquarium (n = 129), Greater Vancouver Zoo (n = 91), and Toronto Zoo (n = 19).

All samples were collected in accordance with approved Animal Care protocols from Laurentian University (2019–02-01, File No.6020970), Toronto Zoo (Project #2021–02-01), Vancouver Aquarium (#2021–01), Greater Vancouver Zoo (Approval #2021–02–18), and the Ministry of Forests, Lands, Natural Resource Operations and Rural Development (Wildlife Act Permit: SU21-618,374).

### Extraction and sequencing

DNA was extracted using a modified protocol from Qiagen DNEasy Blood and Tissue kits adapted to swabs. We evaluated DNA quality on a 1% agarose gel and quantified yield on a Qubit dsDNA HS Assay Kit. Due to low DNA yield, individual samples were then modified to 10 ng/uL for construction of Genotyping by Sequencing (GBS) libraries. All samples below this concentration were considered unsuitable for sequencing (n = 5). Laval University’s genomic analysis platform constructed the GBS libraries (*Pstl/Mspl*) and Génome Québec performed Illumina Novaseq sequencing. Data preparation, genotyping, and filtration were done by Laval University’s genomic analysis platform using STACKS v.2.62^45^ *(denovo* mode), using only forward reads. During bioinformatics treatment, 84 samples were dropped as they were missing more than 15% of genotype calls, were too similar, or had extremely low heterozygosity. We used a minimum genotype coverage of 4, and excluded SNPs which were missing for more than 20% of samples. A total of 5.54 billion reads were demultiplexed and cleaned, translating to an average of 17.2 million reads per sample. Further in the analysis, one Maria sample was removed from the dataset due to questionable origins, leaving 321 samples for final analysis.

### Bioinformatics

Putative bias due to missing data was investigated by performing an identity-by-missingness analysis on the filtered SNPs using PLINK version 1.90b5.3^81^. The resulting multidimensional scaling was represented graphically using sequencing plate number, sample type, population type (zoo *vs.* wild), and source information. No clustering by missingness (a signature of bias) was found (Supplementary Material, Fig. S5).

### Genetic diversity

Genetic diversity was assessed using four different indices within each sampled population as well as within all wild and all zoo individuals. Expected heterozygosity (He), also known as Nei’s gene diversity, represents the likelihood that an individual in a population will be heterozygous at a specific locus. It is used to characterize genetic diversity and is less affected by sample size compared to observed heterozygosity (Ho), which is the actual percentage of heterozygous loci in a population^82^. Wright’s F_IS_ statistic, or inbreeding coefficient, was calculated to directly assess discrepancies between these Ho and He indices^17^. *Vcftools*^83^ was used within each population to calculate individual observed and expected heterozygosity and inbreeding coefficients, with the inbreeding coefficient estimated as (O − E)/(N − E), where O is the number of observed homozygous sites, E is the number of homozygous sites based on Hardy–Weinberg expectation and N is the total number of genotyped loci^76^. The observed and expected heterozygosity (Ho, He) and inbreeding coefficients (F_IS_) per individual frog were then averaged within each population and category described above. Statistical differences between populations and categories were investigated by performing a one-way analysis of variance (ANOVA) or a Kruskal–Wallis test (in the case of non-normal data) with the R package “stats” (v4.1.1^84^). Finally, effective population size was estimated for populations with at least n = 15 using NeEstimator 2.0.1^85^ and the LDNe algorithm^86^ with a lowest allele frequency of 0.1. For comparison, breeding adult population sizes (Nb) were estimated for all wild populations from egg mass surveys in 2021 as 2 × the number of egg masses. This method was determined to be an effective method of estimating Nb for *R. pretiosa*^43^ and is the primary means by which population size is monitored by the *R. pretiosa* recovery team. The Nb for all zoo populations was determined by a count of all sexually mature (≥ 2 years-old) adults in 2021. Loss of heterozygosity through the next 200 generations was inferred from the Ne estimates using the following equation:

Proportion of remaining heterozygosity = ${(1-\left(\frac{1}{2Ne}\right))}^{t}$, where t is the number of generations^20^.

### Genomic differentiation

We investigated the genetic structure among all sampled populations using three approaches. First, a principal component analysis (PCA) was conducted on the individual genotype data using the -pca argument in PLINK. The R package “ggplot2”^84,87^ was used to plot PCA results to investigate any clustering according to 1) population type (zoo *vs.* wild individuals) and 2) genetic source, where genetic source refers to population of origin: a frog caught at Maria has a genetic source of Maria but a frog sampled within a zoo (population of either TZ, VA, or GVZ) would have a genetic source according to where it had been born in the wild or, if zoo-born, its tracked parentage, corresponding to a single wild population or a cross of both parents (ex. Maria or Maria x Elk). Another PCA was performed using only individuals belonging to Maria, Morris, and Elk since global results indicated a clustering pattern mainly driven by those three main wild populations (see results section).

Second, genetic structure among all individuals was investigated with ADMIXTURE 1.3.0^88^ for K ranging from 2 to 15. Cross-validation indices were used to discuss the best values of K across all populations to allow interpretation of the best K in combination with population history (noting a minimum of 11 subpopulations have been recorded historically in Canada^39^). Genetic structure among the wild populations was also investigated running the same ADMIXTURE analysis including only individuals collected from the five wild populations for K ranging from 2 to 7.

Finally, the extent of genomic differentiation was estimated by computing Weir and Cockerham’s Fst a) between zoo and wild categories, and b) among each of the three zoos and the three main wild populations (Maria, Morris, Elk), as well as pairwise tests, all using weir-Fst-pop in *vcftools* version 0.1.17^83^.

## Supplementary Information

Supplementary Information.

## Data Availability

Sequence reads are available on NCBI SRA under Accession: PRJNA982181 and ID: 982181 https://www.ncbi.nlm.nih.gov/bioproject/PRJNA982181
