# Supplementary material for: Continued collaboration of ex situ and in situ programs is critical for the genetic sustainability of the endangered Rana pretiosa
Source: Sci Rep. 2025 May 22;15:17835. doi: 10.1038/s41598-025-01483-4 (PMC12098876; doi:10.1038/s41598-025-01483-4)
Supplement: Supplementary file 1 — Supplementary Information. [file 41598_2025_1483_MOESM1_ESM.pdf]

Continued collaboration of *ex situ* and *in situ* programs is critical for the genetic sustainability of the endangered *Rana pretiosa*

Briar Hunter, Anne-Laure Ferchaud, Eric Normandeau, Kendra Morgan, Arne Mooers, Gabriela Mastromonaco, David Lesbarrères

Supplemental Material

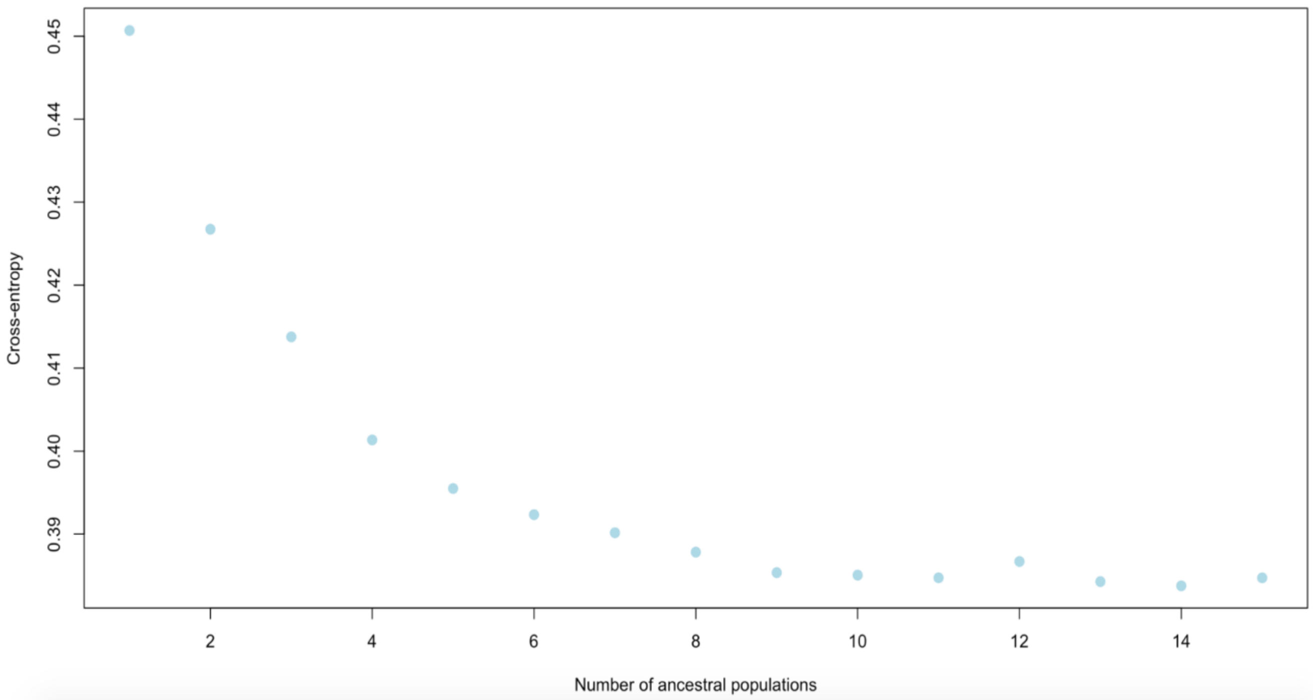

Figure S1. Cross validation for all sampled Oregon Spotted Frogs in Canada (5 wild populations, 3 zoo populations). The lowest cross-entropy value indicates the statistically-optimal number of ancestral populations (K).

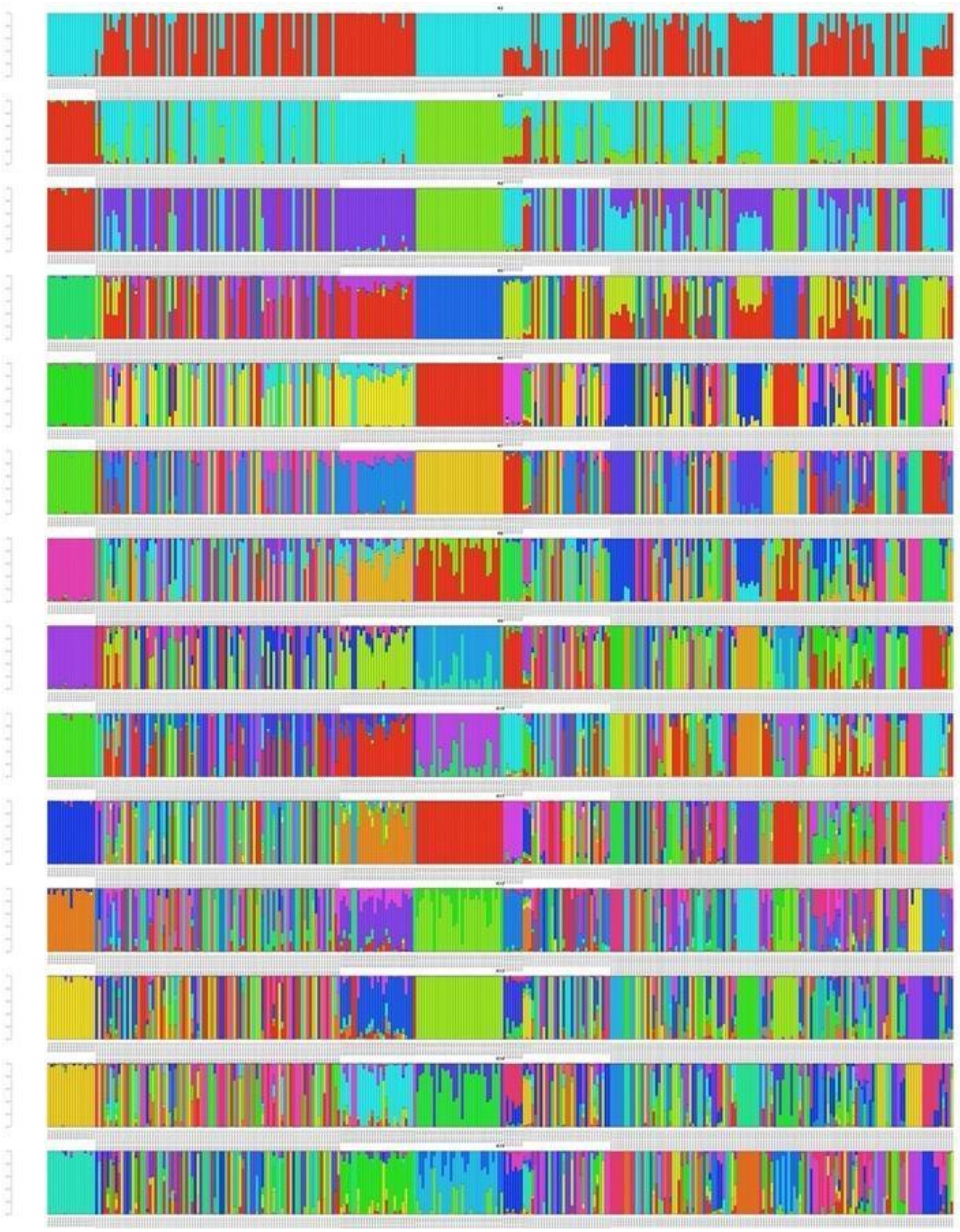

Figure S2. ADMIXTURE from K2 to K15 of all sampled populations of Oregon Spotted Frog (5 wild and 3 zoo populations), ordered by population. Each bar represents one individual frog and colours represent K, the postulated number of ancestral populations.

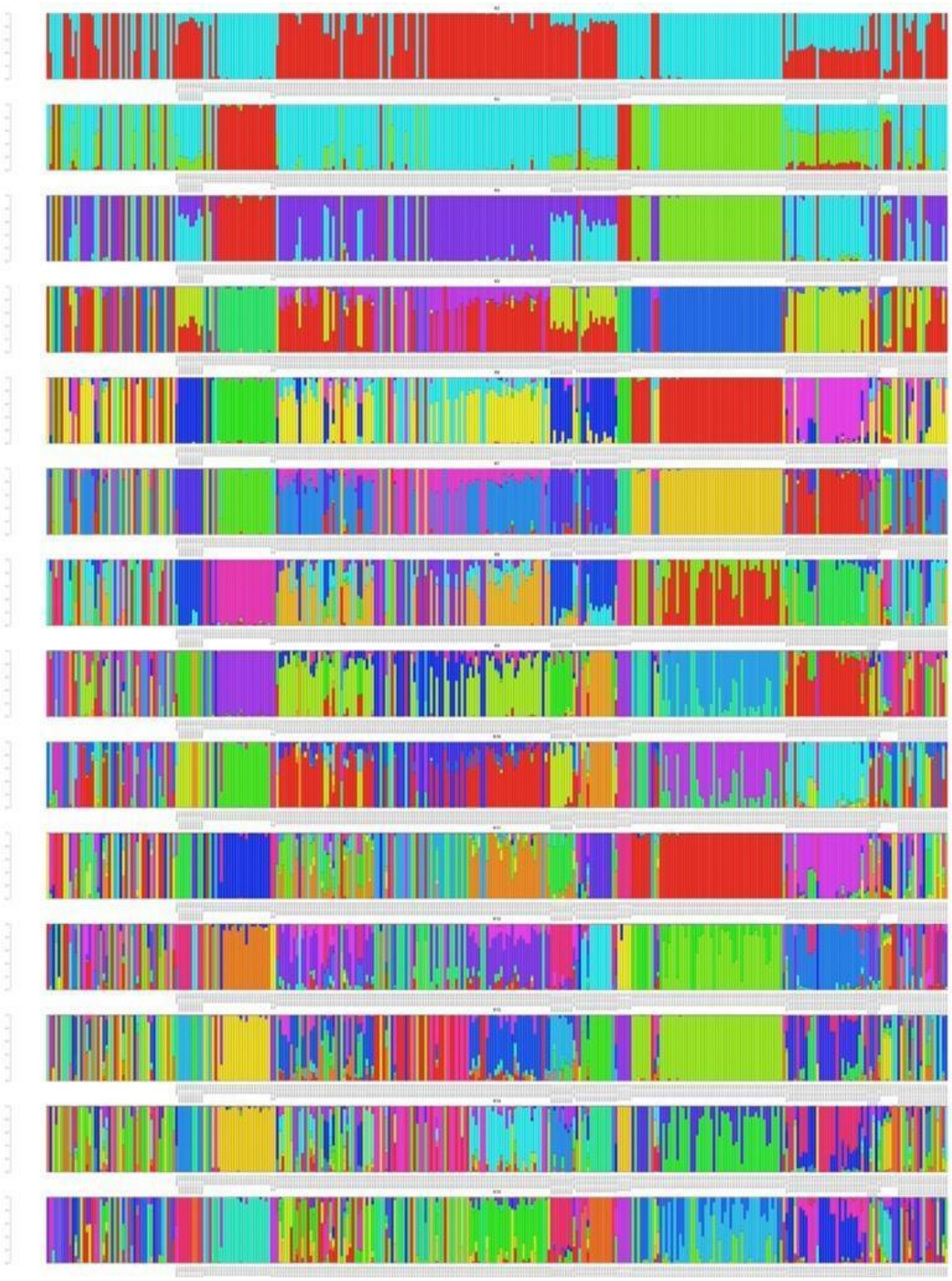

Figure S3. ADMIXTURE from K2 to K15 of all sampled populations of Oregon Spotted Frog (5 wild and 3 zoo populations), ordered by genetic source – the tracked or current lineage of each frog. Each bar represents one individual frog and colours represent K, the postulated number of ancestral populations.

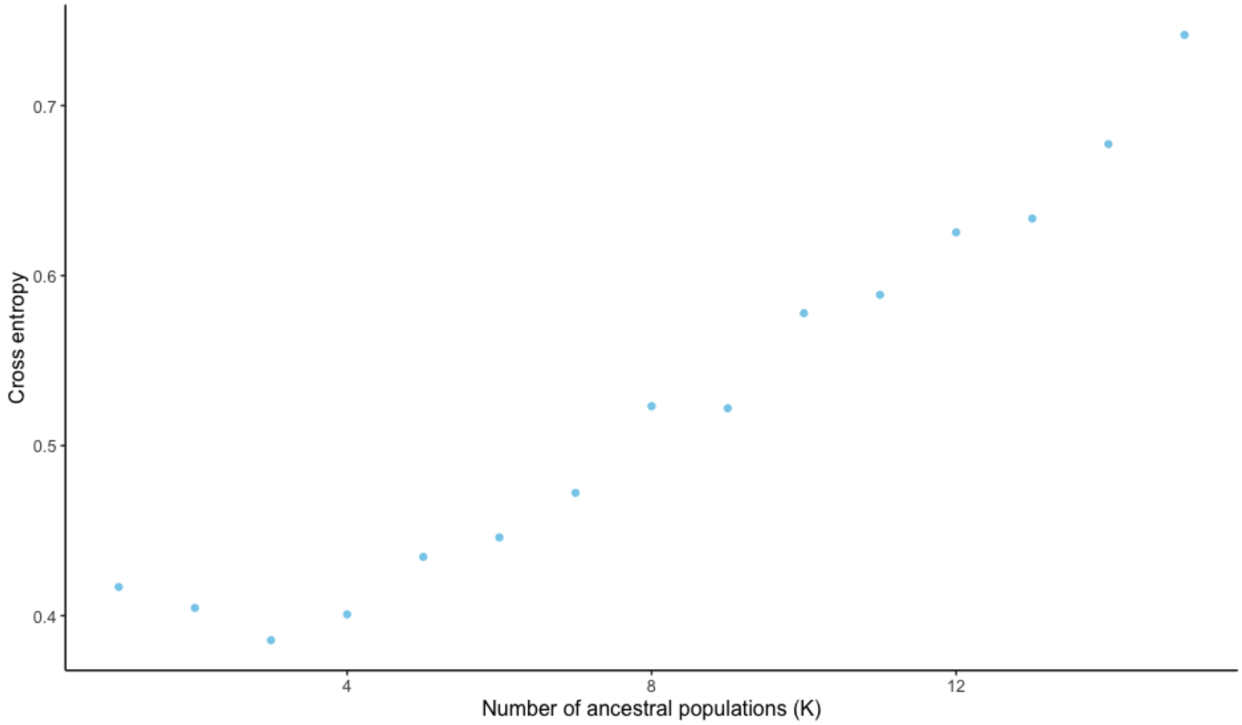

Figure S4. Cross validation for the wild populations of Oregon Spotted Frogs in Canada (MS, MV, MT, EK, ST). The lowest cross-entropy value indicates the statistically-optimal number of ancestral populations (K).

Identity by missingness :

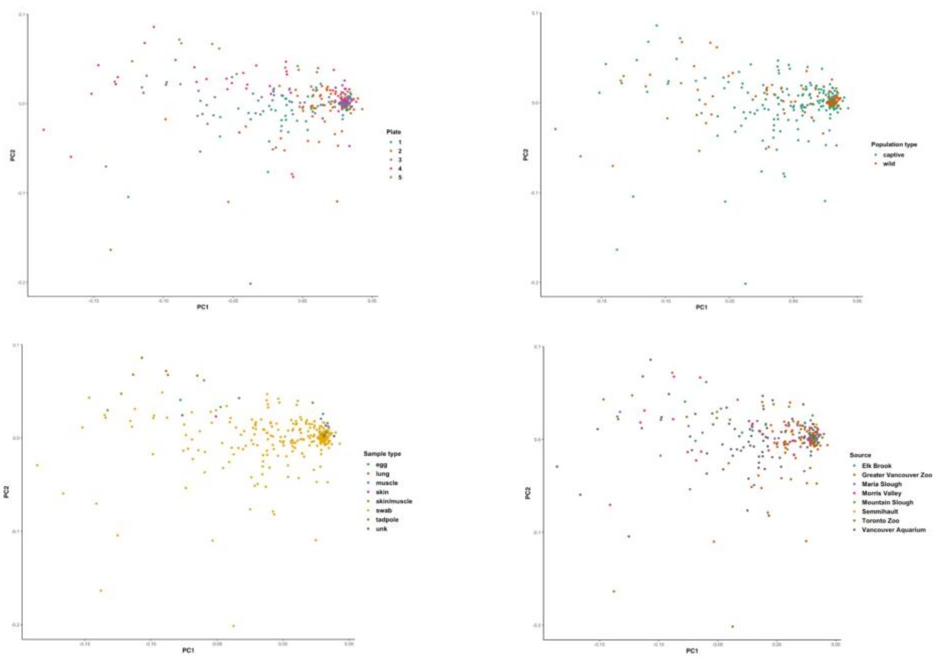

Figure S5. Identity by missingness analysis on the filtered Oregon Spotted Frog SNPs. The data is represented using sequencing plate number, sample type, population type (zoo vs. wild), and source information. No clustering by missingness was found.
